# A New Approach to the Synthesis of Micron-Sized Single-Crystal Silver Plates

**DOI:** 10.3390/mi16101103

**Published:** 2025-09-28

**Authors:** Shilong Li, Xionghui Cai, Aixia Zhai, Tianmeng Zhang, Hui Liu, Jibing Chen

**Affiliations:** 1 School of Chemical and Environmental Engineering, Wuhan Polytechnic University, Wuhan 420023, China; 2 School of Materials Science and Engineering, Huazhong University of Science & Technology, Wuhan 430074, China; 3 School of Mechanical Engineering, Wuhan Polytechnic University, Wuhan 420023, China

**Keywords:** silver plates, single crystal, sulfuric acid, Iron (II) sulfate heptahydrate, wet-chemical

## Abstract

This article describes a novel, non-solvothermal, “citrate-free” method for synthesizing micron-sized, single-crystal silver plates. Transparent hexagonal and truncated triangular single-crystals, mainly enclosed by silver {111} planes, are synthesized by directly adding Iron (II) sulfate heptahydrate solution into silver nitrate solution with appropriate sulfuric acid at an agitation speed of 150 rpm at 8 °C. The mean edge lengths of them range from 4 μm to 16 μm. It is found that the high concentration of silver nitrate and low concentration of Iron (II) sulfate heptahydrate are favorable for the formation of thin silver plates. There was an optimum amount of sulfuric acid and reaction temperature for the preparation of single-crystal silver plates using this method. Sulfuric acid plays an important role in the formation of regular geometric-shaped silver plates. Especially, H ions play the key roles in silver plates to form regular shapes.

## 1. Introduction

Some significant physical and chemical characteristics of silver nanostructures are increasingly demonstrating potential applications in fields such as catalysis [1], localized surface plasmon resonance [2], surface enhanced Raman scattering [3], chemical and biological sensing [4], and diagnosis and therapy of disease [5]. Additionally, these characteristics are related to the shapes of nanostructures. Thus, shape-controlled synthesis of silver nanostructures has attracted great attention.

Much progress has been made in the synthesis of silver nanostructures with different shapes, such as nanocubes [6], nanodisks [7], nanoprisms [8], nanorods [9], and nanowires [10]. Some people also succeed in the synthesis of silver nanoplates. Now, silver nanoplates could be prepared by light-mediated reduction. In these ways, fluorescent light [11], laser [12], and visible light [13,14] could be used as the light source to drive the silver sphere seeds into silver nanoplates. Additionally, the light driving force can also be substituted by the thermal treatment [15,16,17], microwave treatment [18,19,20], and sonochemical treatment [21,22,23]. Silver plates can also be formed at the regulation of chemical reagents, such as cetyltrimethylammonium bromide [24,25] and tannin [26] from seeds in the absence of external driving forces. The ion etching method [27,28] and heat aging [29] can be utilized to convert silver nanoprisms into nanodisks. Kuang presented a synthetic process for producing high-purity silver nanoplates by reducing aqueous silver ions with Ferrocene [30] at the interface between water and organic. Obviously, most of these reported methods have employed citrate or an elevated temperature or other organic compounds to induce the formation of silver nanoplates. However, Sun prepared a silver plate on a semiconductor wafer through a galvanic reaction without any surfactant [31]. Uniform hexagonal silver nanoplate were prepared in the presence of trisodium citrate by choosing light with a selected wavelength used to drive the photochemical growth [32]. Ag nanoplates were prepared through a citrate-free synthesis that involved the reduction of AgNO_3_ by poly(vinyl pyrrolidone) (PVP) in ethanol at 80 °C under a solvothermal condition [33]. It is inconvenient to get the GaAs wafer. There is still a strong need to investigate a new “citrate-free” and non-solvothermal route to synthesize silver plates in the absence of organic compounds. In our previous work [34], the triangular and hexagonal silver plates can be synthesized by a “citrate-free” and non-solvothermal way.

In this paper, the novelty of this paper lies in the fact that for the first time, a “citric acid-free” non-solvent heating method was adopted. Without the need for polymer additives, a solution of ferrous sulfate heptahydrate was directly added to a silver nitrate solution containing an appropriate amount of sulfuric acid, and a single-crystal micrometer-sized silver sheet was prepared by stirring at a speed of 150 revolutions per minute under a temperature of 8 °C. Moreover, the experimental operation method is simple, and the raw materials are readily available. The silver powder prepared by this method will have better conductivity and sintering performance in the electronic manufacturing industry, thereby reducing the cost and energy consumption of electronic manufacturing production, making it more environmentally friendly, and promoting the sustainable development of electronic materials.

## 2. Materials and Methods

In this study, Silver nitrate (AgNO_3_, 99.5%, Analytical reagent, Shanghai Chemical Reagent Co., Ltd., Shanghai, China), Iron (II) sulfate heptahydrate (FeSO_4_·7H_2_O, 99.5%, Analytical reagent, Sinopharm Chemical Regent Co., Ltd., Shanghai, China), sulfuric acid (H_2_SO_4_, 98%, Analytical reagent, Shanghai Chemical Regent Co., Ltd., Shanghai, China), ethanol (C_2_H_5_OH, 99.7%, Analytical reagent, Shanghai Chemical Regent Co., Ltd., Shanghai, China), and deionized water were used as the raw materials without further purification.

In a typical process, 0.02 g silver nitrate and 0.049 g Iron (II) sulfate heptahydrate were dissolved in 20.0 mL and 32.8 mL deionized water, respectively, to prepare the solutions of them. Furthermore, 1.0 mL of sulfuric acid was dissolved in 1000 mL of deionized water to form a sulfuric acid solution with a concentration of 0.0184 mol/L. A total of 1.0 mL of sulfuric acid solution was added to the silver nitrate solution. Then, the Iron (II) sulfate heptahydrate solution was added to the silver nitrate solution immediately at an agitation speed of 150 rpm at 8 °C. Twenty min later, the reaction ceased. After being separated from the mixed solution, the precipitate was washed with deionized water 3–4 times and then ethanol 2–3 times. At last, it was dispersed in 20.0 mL of ethanol to form silver particle suspension solutions for further characterization. Furthermore, we also conducted research on the influence of the synthesis conditions, which include the amount of sulfuric acid, the concentration of silver nitrate, the concentration of ferrous sulfate heptahydrate, the reaction temperature, and the presence of hydrogen ions.

Scanning electron microscopy (SEM) images were obtained using a Hitachi S-3000 microscope (Hitachi, Tokyo, Japan) operated at 15 kV. Transmission electron microscope (TEM) and selected area electron diffraction (SAED) images were measured with a JEM-2100 instrument (JOEL, Tokyo, Japan) operating at 200 kV. Samples for TEM analysis were prepared by dripping a drop of silver nanoparticle suspension solution onto carbon-coated copper grids and drying them in air at room temperature. High-resolution TEM (HRTEM) images were measured by a high-resolution transmission electron microscope operating at 300 kV.

## 3. Results and Discussion

### 3.1. Characterization of the Micron-Sized Single-Crystal Silver Plates

**Figure 1:**
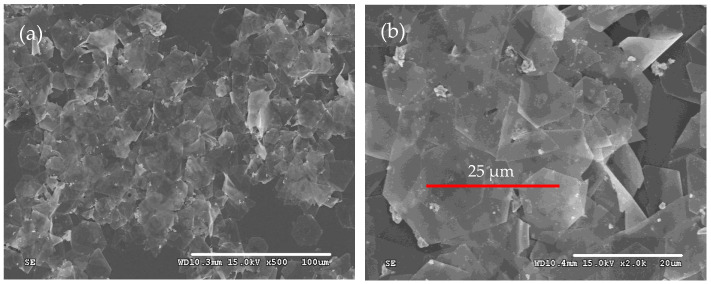
Typical SEM images of silver plates with (**a**) SEM of low magnification and (**b**) SEM of high magnification.

**Figure 2:**
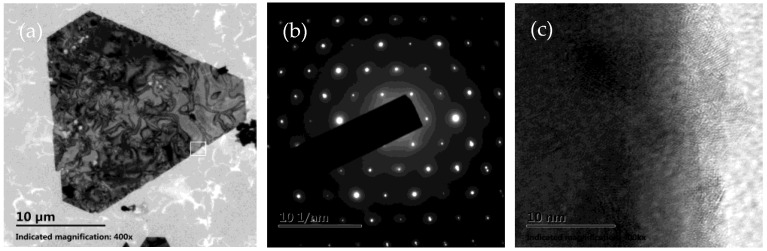
(**a**) TEM image of a single micron-sized silver plate, which was used for the HRTEM and SAED studies. (**b**) SAED pattern of the micron-sized silver plate. (**c**) HRTEM image of the area marked with a white pane in (**a**).

The morphologies of the as-prepared products were first characterized by SEM, as shown in Figure 1. It is obvious that silver microcrystals primarily consist of flat hexagonal and truncated triangular plates (Figure 1a). These plates have smooth surfaces and are so thin (the thickness is less than 10 nm) that they appear transparent. The average size of the plate is approximately 25 μm. The edge lengths of them range from 4 μm to 16 μm (Figure 1b). To get more information about the microstructure of these plates, a typical silver plate was selected for TEM testing. Figure 2a shows a typical TEM image of an individual plate taken from the as-prepared products. Randomly distributed and continuous lines are observed across the face of the flat plate. Gilman believes that these lines may result from the bending of the thin crystal [35]. Figure 2b presents a selected area electron diffraction (SAED) pattern of an individual plate, obtained by directing the incident electron beam perpendicular to the plate. The spot array indicating a hexagonal structure corresponds to the {111} orientation of a silver plate lying flat on the substrate with its top perpendicular to the electron beam [36]. It indicates that the plate is a silver single-crystal structure and that its face is bounded by the {111} planes [37]. Figure 2c shows a high-resolution TEM (HRTEM) image of the edge of an individual silver plate viewed perpendicularly to its side face. The image reveals well-resolved interference fringe patterns with a parallel lattice spacing of approximately 0.23 nm, corresponding to the {111} plane of face-centered cubic silver. Clearly, the parallel fringes of the plate exhibit loose and discontinuous features, indicating that the edge of the silver plate provides active growth sites for attachments of small silver nanoparticles along the {111} plane [38].

The reduction of silver ions by Iron (II) ions can be formulated as follows:

$$Ag^{+}+Fe^{2+}=Ag+Fe^{3+}$$

In the solution, the dissociative sulfate ion acts as not only a stabilizer of silver ions via complex reaction, but also a capping agent of silver colloids for the absorption on their surfaces through the interaction of oxygen and silver atom. As documented in the literature, the {111} plane of silver, having the lowest surface energy, can absorb suitable additives, such as citrate [37,39], which promotes the formation of silver plates with the {111} plane as the basal plane. A sulfate ion has the regular tetrahedron structure with three oxygen atoms that can absorb onto the {111} plane of silver [40]. Therefore, the absorption of sulfate ions on the silver nucleus is crucial for their anisotropic growth. As the crystals grow anisotropically, a single-crystal silver sheet is eventually formed. It can be influenced by the concentration of hydrogen ions. They can compete with silver nuclei and bind with sulfate ions, affecting the anisotropic growth process. Additionally, ferric ions, a byproduct of the reaction and a well-known wet etchant for silver [41] and other noble metals [42], can lead to the anisotropic growth of seeds. They can promote the Ostwald ripening process of silver nuclei into plate structures under the regulation of sulfate ions. This process is also temperature-dependent, with higher temperatures accelerating Ostwald ripening [43]. As the silver plates dissolve and merge, nanoparticles are formed as a byproduct of this dissolution. The growth rate of the silver nucleus is also affected by the concentration of the silver atom, which is affected by the reduction rate. According to the reaction equation, a higher reduction rate leads to a higher concentration of silver atoms [44]. It is beneficial for their diffusion to the existing nuclei. Consequently, a higher reaction rate results in more silver seeds and the formation of thicker plates.

### 3.2. Effect of the Amount of Sulfuric Acid

**Figure 3:**
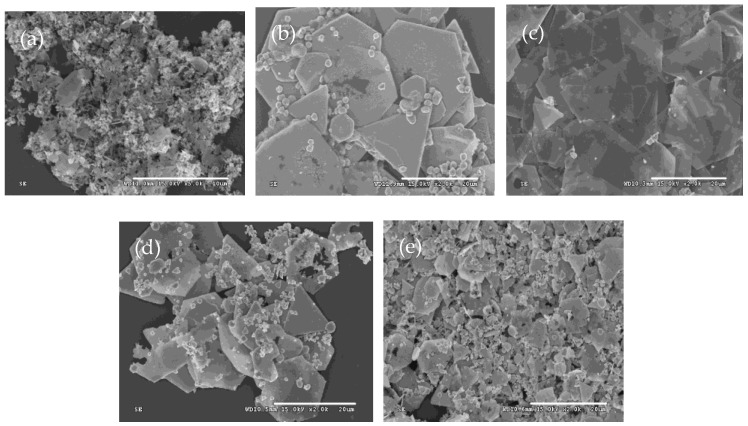
Typical SEM images of silver plates obtained at the amount of sulfuric acid of (**a**) 0 mmol, (**b**) 0.0094 mmol, (**c**) 0.0188 mmol, (**d**) 0.188 mmol, and (**e**) 1.88 mmol.

Similar to some methods mentioned above, additives are essential for promoting the anisotropic growth of silver crystals. In this study, sulfuric acid is the sole additive used throughout the synthesis process. Therefore, the amount of it is critical for the formation of silver plates. To investigate the effect of the amount of sulfuric acid on the morphologies of silver microcrystals, the silver particles were prepared at amounts of sulfuric acid of 0.0 mmol, 0.00092 mmol, 0.0184 mmol, 0.188 mmol, and 1.88 mmol by adjusting their concentrations. All other experimental conditions were identical to the description in Section 2. The SEM images of these resulting products are presented in Figure 3.

The shapes of the silver microcrystals change with the variation of the amount of sulfuric acid. In the absence of sulfuric acid, only a few silver plates with irregular shapes are observed. As the amount of sulfuric acid increases, the proportion of silver plates with hexagonal and truncated triangular shapes increases significantly. When the amount of sulfuric acid is 0.0188 mmol, the silver plates become very thin and appear nearly transparent. However, further increases in it result in a decrease in the percentage of hexagonal and triangular plates and an increase in plate thickness. It reveals that there is an optimum amount of sulfuric acid to produce thin silver plates with regular geometric shapes [45]. When the sulfuric acid is too low, insufficient sulfate ions are absorbed on the silver seeds. The reaction rate is too high due to the poor stabilization of the sulfate ions. So, only a few silver plates are produced (Figure 3a,b). As the concentration of sulfuric acid increases, the regulation of sulfuric acid enhances the anisotropic growth of the silver nuclei. At the same time, the lower reaction rate reduces the concentration of silver seeds, leading to thinner plates (Figure 3c). However, if the amount of sulfuric acid is too large, the reaction rate becomes very low. Also, each face of the silver seeds is covered with sulfate ions. This coverage prevents the seeds from coalescing and growing anisotropically, resulting in the formation of numerous nanoparticles (Figure 3d,e).

### 3.3. The Effect of the Concentration of Silver Nitrate

**Figure 4:**
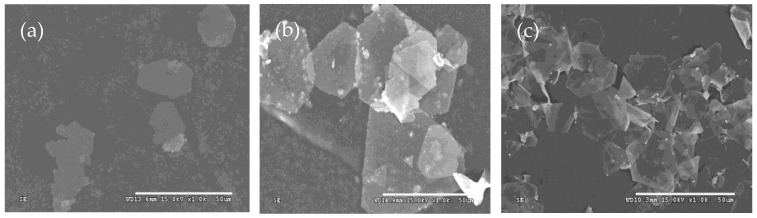
Typical SEM images of silver plates obtained at concentrations of silver nitrate of (**a**) 0.25 g/L, (**b**) 0.5 g/L, and (**c**) 1.0 g/L.

To investigate the effect of the concentration of silver nitrate on the morphologies of as-prepared products, three types of silver microcrystals were prepared at silver nitrate concentrations of 0.25 g/L, 0.5 g/L, and 1.0 g/L. The mole ratios of sulfuric acid and Iron (II) sulfate heptahydrate to silver nitrate were kept constant. The results are shown in Figure 4.

The shape of silver particles changes significantly as the concentration of silver nitrate increases from 0.25 g/L to 1.0 g/L. At the lowest concentration (0.25 g/L), the products mainly consist of a few silver plates and numerous nanoparticles (Figure 4a). As the concentration increases, the proportion of plates rises, and the shapes of these plates become increasingly regular (Figure 4b,c). Specifically, when the concentration is increased from 0.5 g/L to 1.0 g/L, the average edge length of the plates decreases from 30 μm to 20 μm. Additionally, at the higher concentration, the number of nanoparticles decreases significantly, resulting in silver plates that appear transparent and exhibit smooth surfaces. When the concentration of silver nitrate is 0.25g/L, the reaction rate is relatively low, similar to scenarios involving a large amount of sulfuric acid. The silver seeds are so few that they cannot combine to grow into silver plates, and only nanoparticles can be synthesized (Figure 4a). With the increase in the concentration of silver nitrate, the concentration of silver seeds increases, and anisotropic growth happens. In Figure 4b, some nanoparticles are still observed on the silver plates. However, only at sufficiently high concentrations of silver nitrate, the resulting products predominantly consist of flat silver plates (Figure 4c).

### 3.4. Effect of the Concentration of Iron (II) Sulfate Heptahydrate

**Figure 5:**
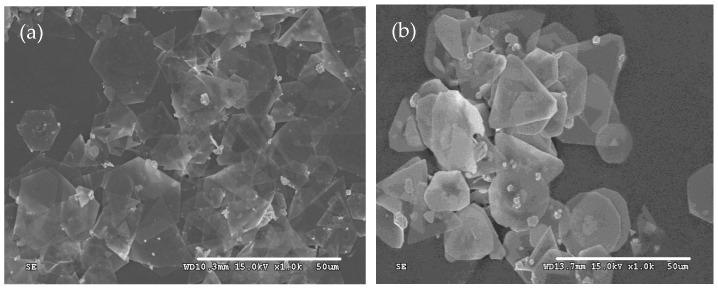
Typical SEM images of silver plates obtained at the concentration of Iron (II) sulfate heptahydrate of (**a**) 1.494 g/L and (**b**) 2.988 g/L.

Similar to Section 3.3, some experiments were conducted to investigate the influence of the concentration of Iron (II) sulfate heptahydrate on the morphologies of silver microcrystals. The mole ratios of sulfuric acid and Iron (II) sulfate heptahydrate to silver nitrate were the same as those described in Section 2. The results are shown in Figure 5.

It is obvious that the concentration of the Iron (II) Sulfate solution significantly influences the morphologies of silver plates. When the concentration of Iron (II) sulfate heptahydrate increases to 2.988 g/L from 1.494 g/L, the thickness of the plates also increases. The increase in the concentration of Iron (II) sulfate heptahydrate leads to a high reaction rate and a greater number of silver seeds, which results in the thicker plates. These findings are consistent with the formation mechanism of silver plates discussed in Section 3.1.

### 3.5. The Effect of the Reaction Temperature

To clarify the behaviors of the silver crystal growth at different reaction temperatures, systematic experiments were conducted at different reaction temperatures. All other conditions were consistent with the description in Section 2.

**Figure 6:**
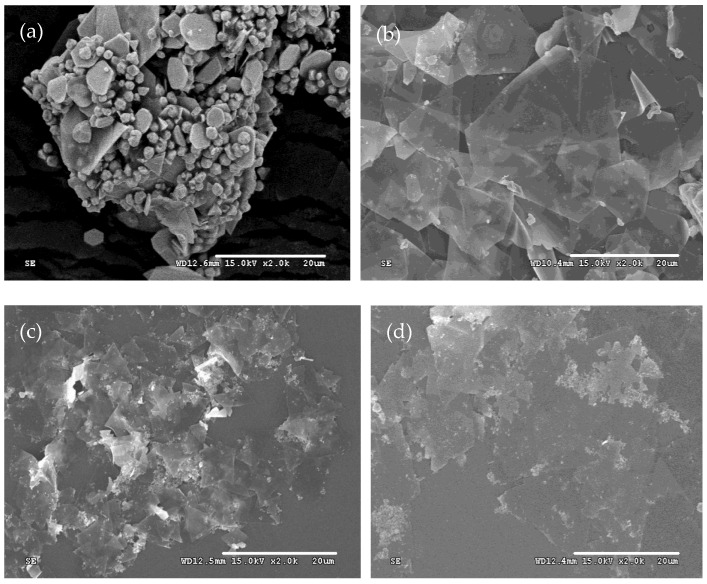
Typical SEM images of silver plates obtained at reaction temperatures of (**a**) 2 °C, (**b**) 8 °C, (**c**) 15 °C, and (**d**) 25 °C.

The results reveal that the morphologies of silver powders change significantly with the increase in the reaction temperature. At 2 °C, the silver microcrystals primarily consist of a few plates and numerous micron-sized particles with the edge length of the plates approximately 4 μm. As the reaction temperature increased to 8 °C, the microcrystals were composed of regular flat plates. Further increases in temperature to 15 °C and 25 °C result in interconnected plates. The size of the plates also increases. Higher temperatures also lead to the formation of lots of nanoparticles. According to the formation mechanism of silver plates, a lower reaction temperature corresponds to a lower reaction rate and a lower concentration of seeds, similar to the description mentioned in Section 3.2 and Section 3.3, resulting in the formation of numerous nanoparticles (Figure 6a). At an optimal reaction temperature, silver plates are predominantly synthesized (Figure 6b). However, if it is so high that the Ostwald ripening becomes prominent, it causes the edges of the plates to merge through this process. Also, at the same time, lots of byproducts, such as nanoparticles (Figure 6c,d), are produced.

### 3.6. Effects of the Hydrogen Ions

It is verified that sulfuric acid plays a crucial role in the formation of a silver plate. Sulfuric acid dissociates into sulfate ion and hydrogen ions in aqueous solution. To clarify the effect of hydrogen ions and sulfate ions on the shapes of silver microcrystals, silver particles were prepared as described in Section 2, except that the sulfuric acid was mixed with sodium sulfate. The mole amount of the sulfate ion was kept the same.

**Figure 7:**
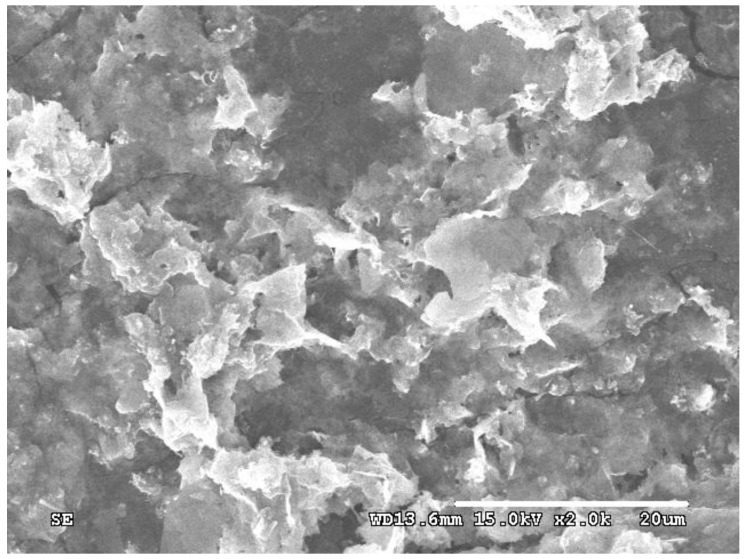
Typical SEM images of silver plates obtained in the presence of 0.0188 mmol Sodium Sulfate.

Compared with the silver particles prepared in Section 2, the morphologies of silver microcrystals changed significantly. The particles primarily consist of irregular and curving silver plates without a clear and smooth edge (Figure 7). It implies that the sulfate ion can promote the growth of silver nuclei into a plate, and the hydrogen ions play a key role in the formation of silver plates with regular shapes. According to the formation mechanism, the hydrogen ions can affect the absorption of the sulfate ion. When sodium sulfate is used instead of sulfuric acid, the hydrogen ions are absent during the synthesis process. On the one hand, it probably affects the spatial structure of the sulfate ion. As mentioned above, the sulfate ion has the regular tetrahedral structure. In the presence of hydrogen ions, they interact with one of the oxygen atoms of the sulfate ion. So, the remaining oxygen atoms are on the same plane and can promote the anisotropic growth of silver seeds. Similar to the effect of citrate, even a small amount of hydrogen ions has a significant effect. In the absence of hydrogen ions, the four oxygen atoms of the sulfate ion are equivalent and have an equal chance to interact with the {111} planes of silver nuclei. However, they cannot align on the same plane, leading to the formation of curved silver plates. Additionally, the Ostwald ripening process may increase due to the complexation of sulfate ions with silver ions, which is caused by the absence of hydrogen ions. Then the irregular and curving silver plates are prepared and connected to each other.

### 3.7. The Discussion of the Formation of Single-Crystal Silver Plates

These experiments mentioned above established that the sulfate ion is the key component in the morphological transformation from spheres into thin plates bounded by {111} facets.

Furthermore, the presence of hydrogen ions could avoid the formation of curving silver plates. The concentration of silver nitrate and Iron (II) sulfate heptahydrate affected the morphologies of the prepared particles through changing the concentration of silver seeds. The Ostwald ripening increases with the increase in reaction temperature. All these elements had an influence on the shapes of the particles.

**Figure 8:**
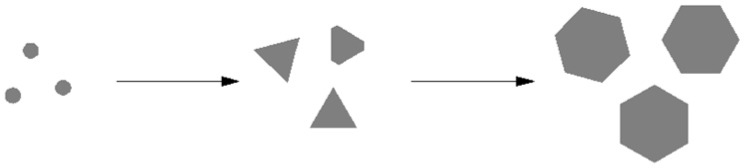
The sketch map of the formation mechanism of plate silver microcrystals.

The formation mechanism of plate silver microcrystals can be described in Figure 8. It concludes that the formation process of silver micro-sized plates involved three steps: the formation of solid spherical silver particles, the transformation of spherical particles to triangular crystals, and lastly the production of hexagonal and truncated triangular single-crystals. It is similar to that of the silver nanoplates [46,47,48].

## 4. Conclusions

In this paper, the triangular and hexagon silver plates were synthesized by a “citrate-free” and non-solvothermal way. The effect of reaction conditions on the morphologies of the prepared silver particles was investigated. The conclusions are shown as follows.

(1) Micron-sized single-crystal silver plates can be synthesized by directly adding Iron (II) sulfate heptahydrate solution into silver nitrate solution containing a suitable amount of sulfuric acid at an agitation speed of 150 rpm at 8 °C, and the face of the plate was bounded by the {111} planes.
(2) Sulfuric acid plays a key role in the formation of silver plates with regular geometric shapes. A sulfate ion can promote anisotropic growth of silver seeds. Hydrogen ions are essential for the formation of silver plates with regular geometrical shapes.
(3) There are optimal conditions for the reaction temperature and the amount of sulfuric acid required to synthesize single-crystal silver plates in the presented way. Too low a reaction rate is not beneficial for the formation of silver plates. Additionally, the thickness of plates increases with the increase in concentration of Iron (II) sulfate heptahydrate. At sufficiently high reaction temperatures, the plates will merge through the Ostwald ripening process.

In the future, we will apply the prepared silver sheets to the research on conductive adhesives and sintering silver in the field of electronic manufacturing, and explore its feasibility.

## Data Availability

The original contributions presented in the study are included in the article; further inquiries can be directed to the corresponding author.
